# Pre-inhalation of hydrogen-rich gases protect against caerulein-induced mouse acute pancreatitis while enhance the pancreatic Hsp60 protein expression

**DOI:** 10.1186/s12876-021-01640-9

**Published:** 2021-04-19

**Authors:** Kun Li, Hongling Yin, Yi Duan, Peizhen Lai, Yancheng Cai, Youzhen Wei

**Affiliations:** 1 Department of Pathophysiology, Tongji University School of Medicine, 1239 Siping Road, Shanghai, 200092 China; 2 Shanghai Asclepius Meditec Co. Ltd., 758 Jiaxin Road, Shanghai, 201818 China; 3 Research Center for Translational Medicine and Key Laboratory of Arrhythmias of the Ministry of Education of China, East Hospital, Tongji University School of Medicine, 150 Jimo Road, Shanghai, 200120 China

**Keywords:** Acute pancreatitis, Hydrogen-rich gases, Hsp60, Inflammation, Oxidative stress

## Abstract

**Background:**

Acute pancreatitis (AP) lacks targeted prevention and treatment measures. Some key points in the pathogenesis of AP remain unclear, such as early activation of pancreatic enzymes. Several recent reports have shown the protective effect of hydrogen on several AP animal models, and the mechanism is related to antioxidant activity. Heat shock protein 60 (Hsp60) is known to accompany pancreatic enzymes synthesis and secretion pathway of in pancreatic acinar cells, while role of hsp60 in AP remains a topic. Aim of this study was to investigate effect of hydrogen pretreatment on AP and the mechanisms, focusing on pancreatic oxidative stress and Hsp60 expression.

**Methods:**

80 mice were randomly assigned into four groups: HAP group, AP group, HNS group, and NS group and each group were set 3 observation time point as 1 h, 3 h and 5 h (n = 6–8). Mouse AP model was induced by intraperitoneal injection of 50 μg/kg caerulein per hour for 6 injections both in AP and HAP groups, and mice in NS group and HNS group given normal saline (NS) injections at the same way as control respectively. Mice in HAP group and HNS group were treated with hydrogen-rich gases inhalation for 3 days before the first injection of caerulein or saline, while mice in AP group and NS group in normal air condition. Histopathology of pancreatic tissue, plasma amylase and lipase, plasma IL-1 and IL-6, pancreatic glutathione (GSH) and malondialdehyde (MDA), and Hsp60 mRNA and protein expression were investigated. Comparisons were made by one-way analysis of variance.

**Results:**

The pancreatic pathological changes, plasma amylase and lipase activity, and the increase of plasma IL-1 and IL-6 levels in AP mice were significantly improved by the hydrogen-rich gases pretreatment, Meanwhile, the pancreatic GSH content increased and the pancreatic MDA content decreased. And, the hydrogen-rich gases pretreatment improved the Hsp60 protein expression in pancreatic tissues of AP mice at 1 h and 5 h.

**Conclusions:**

Pre-inhalation of hydrogen-rich gases have a good protective effect on AP mice, and the possible mechanisms of reduced oxidative stress and the early increased pancreatic Hsp60 protein deserve attention.

## Background

Acute pancreatitis (AP) is an acute inflammatory disorder of the pancreas with a high morbidity and mortality, and the damages may be local, or accompanied with systemic inflammatory response syndrome and distant organ dysfunction. The mechanism of AP has been a focus of concern for a long time. Now it is generally accepted that the abnormal activation of pancreatic enzymes in pancreatic acinar cells, and the inflammatory mediators and cytokines play important roles in the occurrence and development of AP, but many detailed events remain unclear [1]. Up till now, there has been a lack of targeted prevention and control measures for the key pathogenesis of AP [2].

Heat shock protein 60 (Hsp60), known as molecular chaperonin, is a member of heat shock protein family which functions in protein folding, transmembrane translocation, assembly, and disassembly. In pancreas, there is evidence that Hsp60 coexists with pancreatic zymogen granules along the exocrine secretion pathway in pancreatic acinar cells [3], and studies suggest that the decreased expression of Hsp60 in pancreas may correlate with the occurrence of AP in rat [4, 5]. The protective effect of HSP60 on AP is to be discussed further.

As the lightest gas molecule, hydrogen gases is no longer considered a biological inert gas, reportedly an anti-oxygen free radical with anti-inflammatory effects, and is expected to have good prospects in medical application [6]. Several recent studies have found that hydrogen gas or hydrogen-rich saline have a protective effect on experimental AP and mechanisms are being explored [7–9].

In the present study, we intent to investigate the effect of pre-inhalation of hydrogen-rich gases on AP mice induced by caerulein, and the possible mechanisms. For which, in addition to the level of oxidative stress in pancreatic tissues, we pay more attention to the alteration of pancreatic Hsp60 expression before and after the hydrogen-rich gases treatment. Our goal is to reveal the possible links of Hsp60 protein in the protective effects of hydrogen pretreatment on AP mice.

## Methods

### Animals

At least 80 adult C57Bl/6 mice weighing 22 ± 2 g, male or female, purchased from Shanghai SLAC Laboratory Animal Co. Ltd. were used in this experiment. The animals were housed under standard conditions at a 12:12 h light–dark cycle, with free access to water ad libitum and lab chow at 23 ± 2 °C and 55 ± 5% humidity. Same sex litter mates were housed together in individually ventilated cages with two or four mice per cage, and the animals’ welfare and the experimental procedures were approved by the Medical and Life Sciences Ethics Committee of Tongji University, Shanghai, China.

### Establishment of AP mouse model and experimental protocol

Experimental mouse AP model was induced by caerulein (Sigma Chemical, St. Louis, USA) in the same way as a previous study [10]. In brief, mice fasted for 12 h before receiving intraperitoneal injections (i.p.) of 50 μg/kg caerulein hourly, totaling six injections to induce mouse AP model, and mice received normal saline (NS) i.p. instead of caerulein as control.

C57Bl/6 Mice were randomly assigned into the following 4 groups: NS group, HNS group, AP group and HAP group, and each group were set 3 observation time point as 1 h, 3 h and 5 h, and at each time point n = 6 – 8 for each group (“n” indicate the total number of mouse used and mice died during model replication were excluded). Mice in AP group and HAP group both received the caerulein injections and mice in NS group and HNS group both received the NS injections as above mentioned. But mice in HAP group and HNS group were treated with hydrogen-rich gases (42% H_2_ + 21% O_2_ + 37% N_2_) inhalation for 3 days before the first injection of caerulein or saline, while mice in AP group and NS group in normal air condition. 1 h, 3 h or 5 h after the final injection of caerulein or NS, mice were euthanized under isoflurane anesthesia. Blood samples were taken, placed in the heparin-treated tubes, centrifuged immediately at 3000 rpm for 10 min, and the plasma was collected and stored in liquid nitrogen. Meanwhile, the pancreas tissue were dissociated, rinsed and divided to 3 parts. The head portions of the pancreas were fixed in 4% paraformaldehyde for pathological evaluation, and the rest of the pancreatic tissues placed in empty tubes or in tubes with 0.5 ml RNAiso Plus (Takara Bio, Beijing, China). Both were stored in liquid nitrogen for later usage.

### Histopathological analysis

The pancreatic tissues fixed in the 4% paraformaldehyde above were paraffin embedded, sliced (5 μm), and stained by hematoxylin and eosin (HE). The slides were observed and studied under a high power microscope. At least 15 randomly chosen microscopic fields from three slides of three mice in every group were examined and evaluated blindly. Images were captured by digital image analysis system Motic Med 6.0 (Motic Software Engineering Co. Ltd., Xiamen, China). Pancreatic histopathological changes were given scores as described previously [10], Scoring for (i) The interstitial and acinar edema: 0 absent, 1 focal expansion of interlobular septa, 2 expanded expansion of interlobular septa, 3 focal expansion of intralobular septa, 4 expanded expansion of intralobular septa; (ii) the presence of vacuolization: 0 absent, 1 less than 20%, 2 20% to 35%, 3 35% to 50%, 4 more than 50%; (iii) the interstitial infiltration of neutrophils or lymphocytes: 0 absent, 1 1 to 10 /high power field (HP), 2 11 to 20 /HP, 3 20 to 30 /HP, 4 > 30 /HP; (iv) the necrosis of acinar cell: 0 absent, 1 1 to 5 /HP, 2 5 to 10 /HP, 3 11 to 15 /HP, and 4 > 15 /HP. In each group, means of the scores in edema, vacuolization, infiltration and necrosis were presented as results of the final pathological scores.

### Measurement of plasma amylase and lipase activity

The activity of plasma amylase and lipase were determined using the respective enzymatic reaction commercial kits (Jiancheng Technology, Nanjing, China). Methods were according to the manufacturer’s instructions (Cat No. C016 and Cat No. A054-1-1), samples were mixed, water bath, optical density (OD660nm and OD420 nm) detection, and plasma amylase and lipase activity were calculated according to formula provided on the instruction. Result of the amylase activity was presented as unit per deciliter (U/dL), and the lipase activity was presented as unit per liter (U/L).

### Assay for plasma IL-1 and IL-6

Plasma interlukin-1 (IL-1) and IL-6 levels were quantified by mouse IL-1β or IL-6 commercial available enzyme-linked immune-sorbent assay (ELISA) kits (R&D Systems, Abingdon, UK). Protocols were according to the manufacturer’s instruction of the corresponding kits, and each specimen was measured twice. Levels of IL-1and IL-6 in plasma were calculated as pg/ml, and the results presented as a percentage of the NS group of 1 h (the NS1h group).

### Measurement of pancreatic GSH and MDA

The pancreatic tissues in empty tube stored in liquid nitrogen were thawed on ice and homogenized in NS. Part of the homogenate was used for protein quantification by the use of Pierce BCA Protein Assay Kit (Thermo, Rockford, USA). 0.1 ml of the homogenate was centrifuged at 3500 rpm, 4 °C for 10 min, and the supernatant used for the reduced glutathione (GSH) detection. The content of GSH in pancreas was determined by commercial GSH Assay Kits (Jiancheng Technology, Nanjing, China) which contain total glutathione (T-GSH) and oxidative glutathione (GSSG) standard and based on dithio-bis-nitrobenzoic acid (DTNB) cycle reaction. Method was according to the instruction of the manufacturer (Cat No. A061-1) and content of GSH calculated by T-GSH minus double GSSG according to the instruction. Data was calculated and presented as μmol per gram of total protein (μmol /g).

0.1 ml of the homogenates was used for the malondialdehyde (MDA) detection. The content of MDA in pancreas was determined by commercial MDA Assay Kits (Jiancheng Technology, Nanjing, China) based on thiobarbituric acid (TBA), and method was according to the instruction of the manufacturer (Cat No. A003-1). Data was calculated and presented as nano-mole per milligram of total protein (nmol /mg).

### Immunohistochemistry

The expression of in situ Hsp60 protein in pancreas was detected by immunohistochemistry, with the same protocol as the former [10]. Slides of the paraffin section of mouse pancreatic tissue dewaxed, hydrated, and heat-mediated antigen retrieval, and then blocked in 5% bovine serum albumin (BSA) (Sangon Biotech, Shanghai, China) for 1 h at room temperature. The slides were subsequently incubated with rabbit anti-Hsp60 antibody (1:600 diluted) (Enzo, Pennsylvania, USA) overnight at 4 °C, and horse radish peroxidase (HRP)-conjugated secondary antibody (Beyotime, Shanghai, China) for 2 h at room temperature. Finally, the slides were stained with diaminobenzidine (DAB) (Sangon Biotech, Shanghai, China) and hematoxylin.

Positive signals with brownish staining were observed under microscopy. Images were captured by Motic Med 6.0 (Motic Software Engineering Co. Ltd., Xiamen, China), and the optical density (OD) of the positive signals was measured semi-quantitatively by the software of Image J. Results were presented as a percentage of the NS1h group.

### Western blotting

The expression of Hsp60 protein in pancreatic tissue was detected by Western blotting as well, and the protocol was similar to that of the former [10]. Mouse pancreatic tissues preserved in liquid nitrogen were thawed and homogenized, and the total protein was extracted. Protein quantification was carried out with the Pierce BCA Protein Assay Kit (Thermo, Rockford, USA). Protein samples loaded and separated on 10% SDS-PAGE gels was transferred to PVDF membrane. The PVDF membrane was blocked in 5% BSA (Sangon Biotech, Shanghai, China) for 2 h at room temperature, and subsequently incubated with rabbit anti-Hsp60 antibody (1:1000 diluted) (Enzo, Pennsylvania, USA) or β-actin antibody (1:2000 diluted) (Cell Signaling Technology, Boston, USA) overnight at 4 °C, and a HRP-conjugated secondary antibody (1:2500 diluted) (Beyotime, Shanghai, China) for 1 h at room temperature. Positive signals in membrane were detected by chemiluminescence with the use of ECL reagent (Beyotime, Shanghai, China) and the integrated density (IntDen) of the positive signals was analyzed by the software of Image J. Results was calculated as a relative expression of Hsp60 protein adjusted to the corresponding β-actin expression, and data was shown as a percentage of the NS1h group.

### Quantitative real time PCR

The expression of Hsp60 mRNA in pancreas was detected by real-time quantitative PCR (RT-PCR), and protocols were similar to that of the former [4]. Mouse pancreatic tissues in tubes with RNAiso Plus (Takara Bio, Beijing, China) stored in liquid nitrogen were thawed and homogenized, and total RNA was extracted. 1 g of RNA was reverse transcribed to single-stranded complementary DNA (cDNA) according to the manufacturer’s recommended procedure of the HiScript III RT SuperMix Kits (Vazyme, Nanjing, China). Next, quantitative RT-PCR was performed on QuantStudio 6 Flex System (Therm Lifetech, Walsham, USA) by using Power SYBRTM Green PCR Master Mix (Thermo Fisher, Walsham, USA), with GAPDH as the house-keeping gene, and the annealing temperature was 58 °C and the number of PCR cycles was chosen to stop the reaction in the linear phase of amplification (40 cycles). Primer sequences were used as follow: hsp60, forward (5′-CAGTGAAGGATGGAAAAACCCT-3′) and reverse (5′-TCTTTGGTGACAATGACCTCC C-3′); GAPDH, forward (5′-GCATCTTCTTGTGCAGTGCC-3′) and reverse (5′-TACGGCCAAATCCGTTCACA-3′). Relative quantification was calculated by the double ΔCt method, and results were present as a percentage of the NS1h group.

#### Statistical analysis

Data analysis was performed using statistical software SPSS version 22.0. All results were expressed as mean ± standard error of the mean (SEM), Comparisons were made by one-way analysis of variance (ANOVA) followed by the Bonferroni post hoc test. Values of *P* < 0.05 were considered statistically significant.

At least 6 mouse models were constructed at each time point in each group. The number of observed animals per indicator varies according to source of specimen and the method used and for each indicator the number of samples in each group of the test indexes is equal. For pathological evaluation, at least 15 randomly chosen microscopic fields from three slides of three mice in every group were examined and evaluated blindly. Plasma amylase and lipase activity, pancreatic GSH and MDA content which detected by biochemical methods as well as IL-1 and IL-6 which detected by ELISA each with animal number 6, the included animal number n is 6. Molecular biology indicators such as Hsp60 protein and mRNA expression which detected by immunohistochemistry, Western blotting or RT-PCR, each contain at least 3 animals.

## Results

### Histopathological scores and evaluation of pancreatic tissue

**Fig. 1:**
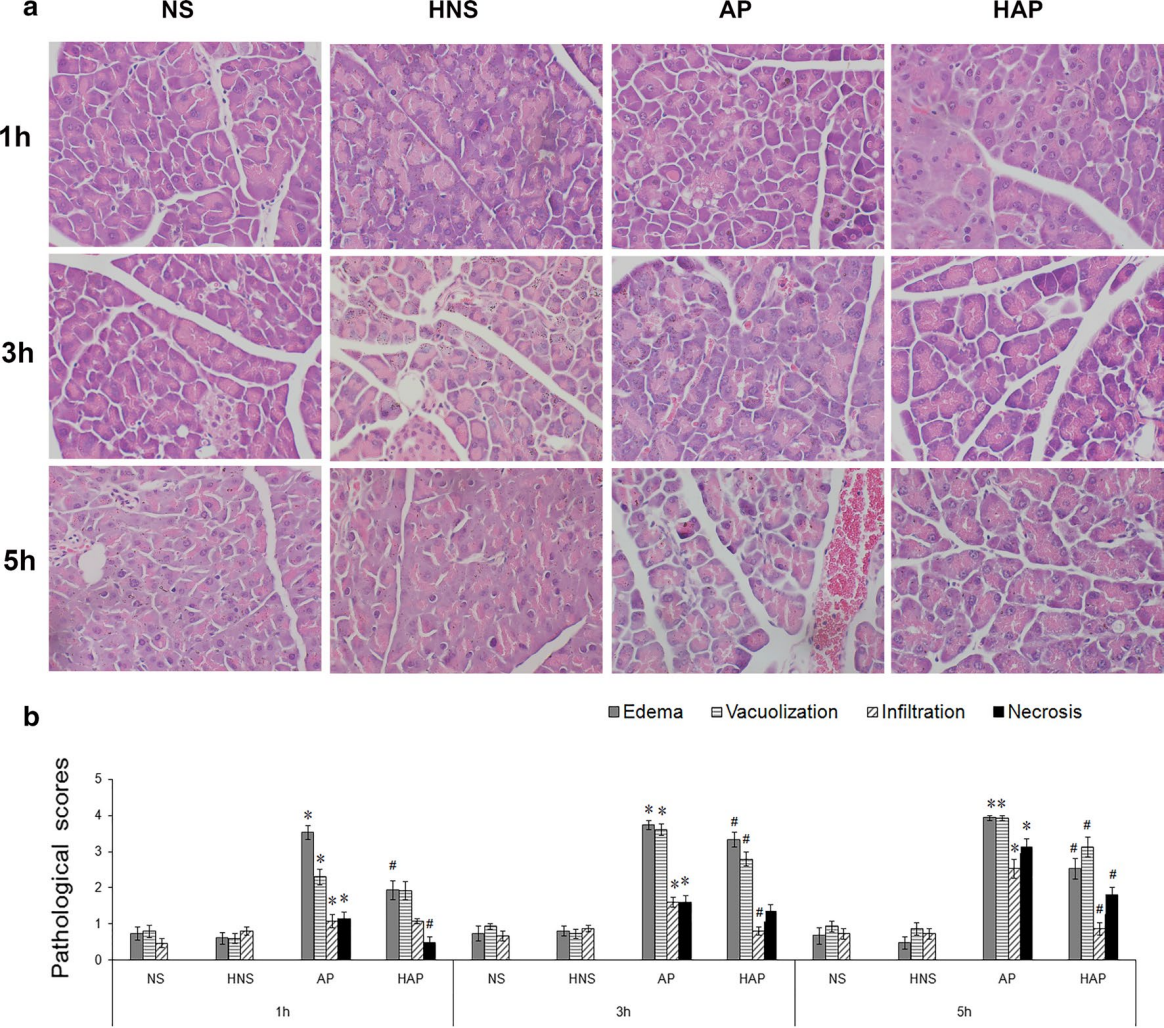
Effects of H_2_ pre-inhalation on pancreatic pathology (HE, × 400). **a** Representative pathological images of mouse pancreatic tissue. Acute pancreatitis (AP) model was induced by intraperitoneal injection of 50 μg/kg caerulein per hour for 6 injections in C57Bl/6 mice, and mice given normal saline (NS) injections at the same way as control. Some mice were treated with hydrogen-rich gases (42% H_2_ + 21% O_2_ + 37% N_2_) inhalation for 3 days before the first injection of caerulein or saline, while others still in normal air condition. NS: control mouse, HNS: control mouse given treatment of the hydrogen-rich gases pre-inhalation, AP: mouse with AP, HAP: mouse with AP and treated with the hydrogen-rich gases pre-inhalation; b, Pathological scores for edema, vacuolization, inflammation, and necrosis in each group. At least 15 randomly chosen microscopic fields from three slides of three mice in every group were examined and evaluated blindly. Results are presented as means ± SEM (n = 6). *p < 0.05 compared with the NS group at the same time point; ^#^*p* < 0.05 compared with the AP group at the same time point

The pathological changes of the pancreas in mice of AP group were gradually aggravated over time, and treatment of the hydrogen-rich gases (42% H_2_ + 21% O_2_ + 37% N_2_) inhalation improve the pathological changes in AP mice significantly to varying degrees, as shown in Fig. 1. Figure 1a represented the typical pathological changes of the pancreas, whereas the pathological scores of the pancreas including edema, vacuolization, infiltration, and necrosis were shown in Fig. 1b. 1 h after the modeling, interstitial edema, moderate vacuolization and infiltration were observed, and at 3 h, in addition to edema, severe vacuolization, congestion and infiltration were observed. The pancreatic lesions were developed further at 5 h with intra-lobular septa expansion, obvious congestion and infiltration, and scattered necrosis of the acinar cells. When compared with the AP group, the pathological changes of pancreas in HAP group were improved significantly at each time point. However, there was no significant difference in the pathological changes of mice in the NS group and the HNS group at the same time point.

### Activity of amylase and lipase in plasma

**Fig. 2:**
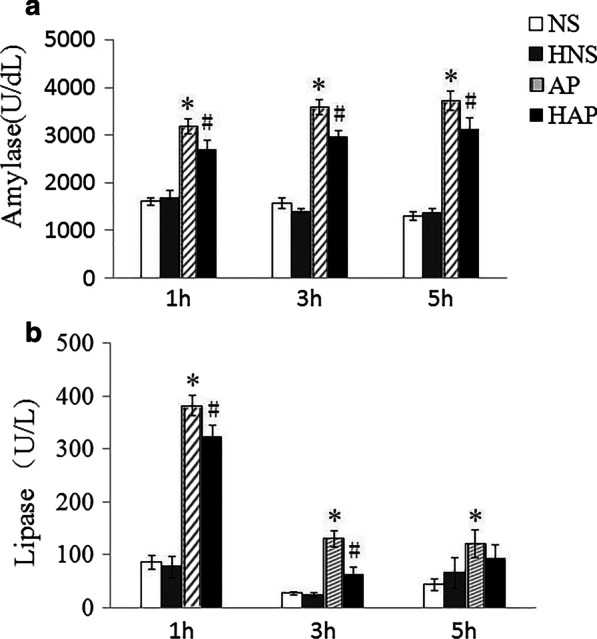
Effects of H_2_ pre-inhalation on plasma amylase and lipase activity. **a** Amylase activity in plasma; **b** Lipase activity in plasma. Results are presented as means ± SEM (n = 6). *p < 0.05 compared with the NS group at the same time point; ^#^*p* < 0.05 compared with the AP group at the same time point

The activity of plasma amylase and lipase in each group of mice was shown in Fig. 2. As shown in Fig. 2a, the activity of plasma amylase in AP group was significantly higher than that in NS group (*p* < 0.05), and that in HAP group was significantly lower than that in AP group at each time point (*p* < 0.05). There was no difference in the activity of plasma amylase in the NS and the HNS group at the same time point (*P* > 0.05).

As shown in Fig. 2b, the activity of plasma lipase in AP group was significantly higher than that in NS group at each time point (*p* < 0.05), and that in HAP group was significantly lower than in AP group at 1 h and 3 h (*p* < 0.05). There was no difference in the activity of plasma lipase in the NS and the HNS group at the same time point (*P* > 0.05).

### Levels of IL-1 and IL-6 in plasma

**Fig. 3:**
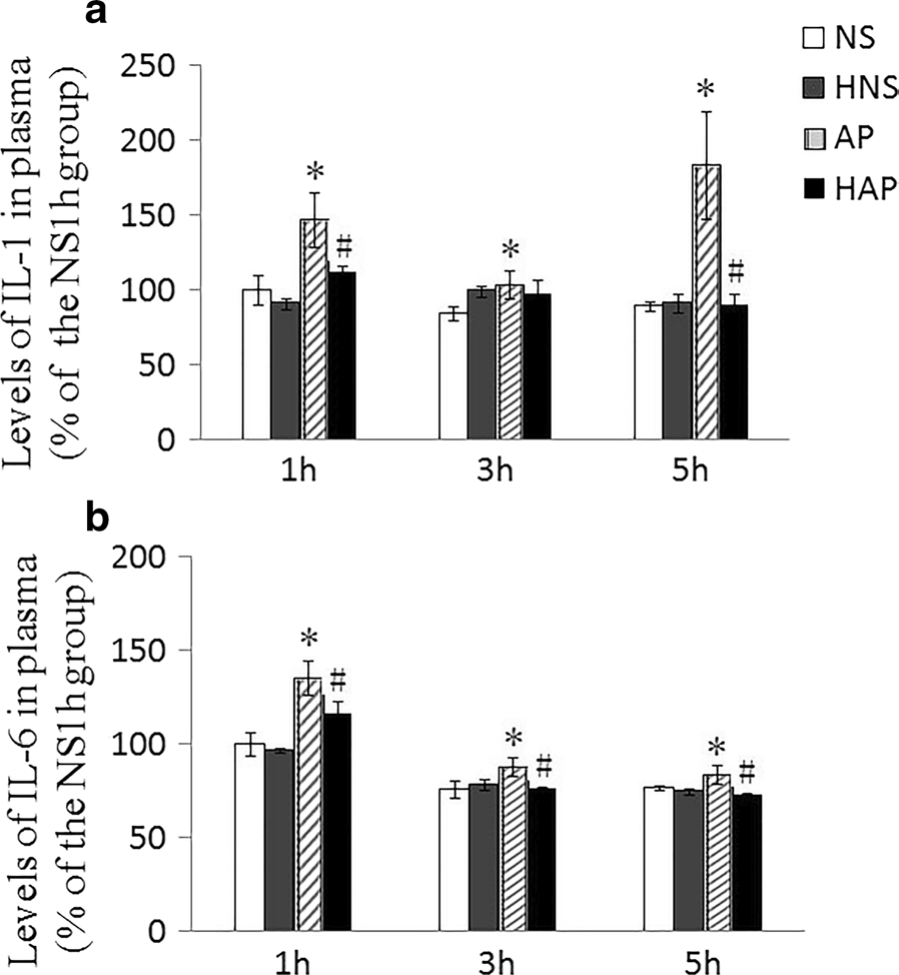
Effects of H_2_ pre-inhalation on plasma IL-1 and IL-6 levels. **a** IL-1 levels in plasma; **b** IL-6 levels in plasma. Results are presented as means ± SEM (n = 6). *p < 0.05 compared with the NS group at the same time point; ^#^*p* < 0.05 compared with the AP group at the same time point

Plasma IL-1 and IL-6 levels in each group of mice were shown in Fig. 3. At each time point, both plasma IL-1 and IL-6 levels in AP group were significantly higher than those of NS group (*p* < 0.05), and the IL-1 levels in HAP group were lower than those of NS group significantly at 1 h and 5 h (Fig. 3a), while the IL-6 levels in HAP group lower than those of NS group significantly at each time point (Fig. 3b). At the same time point, there were no significant difference in plasma IL-1 and IL-6 levels between the NS group and the HNS group (*P* > 0.05).

### Content of GSH and MDA in pancreatic tissue

**Fig. 4:**
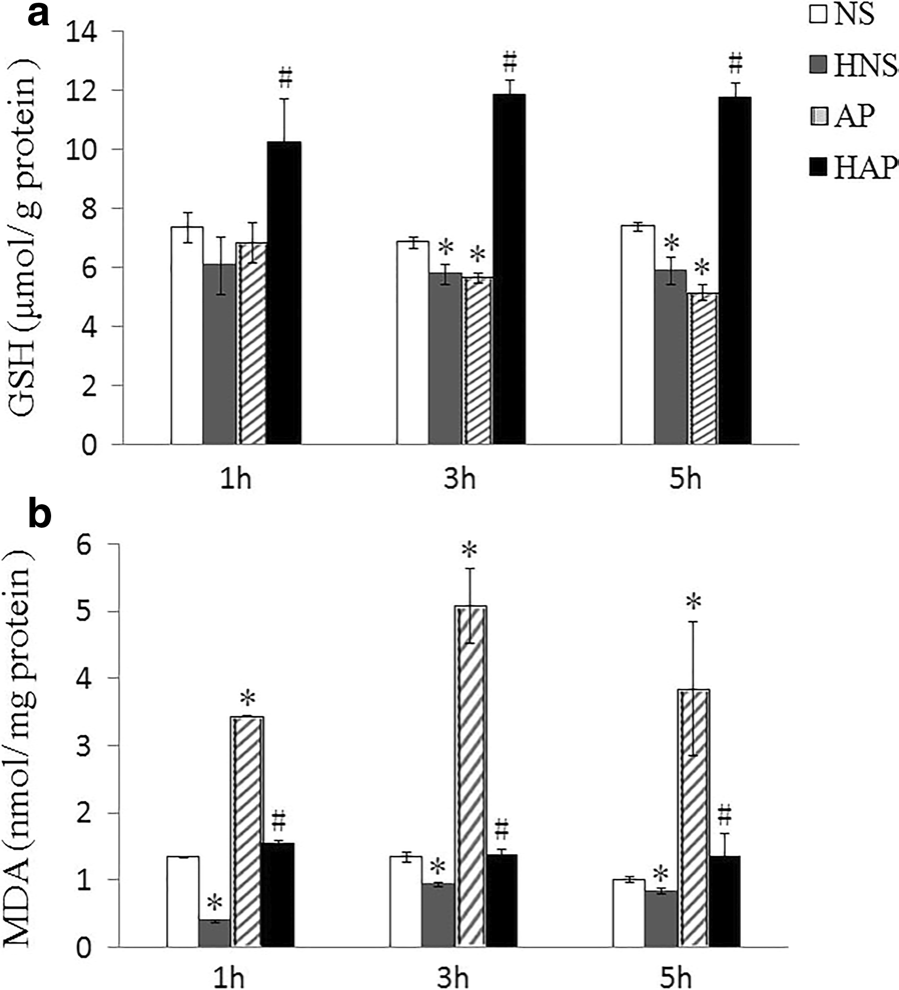
Effects of H_2_ pre-inhalation on pancreatic GSH and MDA content. **a** GSH content in pancreatic tissue; **b** MDA content in pancreatic tissue. Results are presented as means ± SEM (n = 6). *p < 0.05 compared with the NS group at the same time point; # p < 0.05 compared with the AP group at the same time point

As shown in Fig. 4a, when compared with the NS group, the reduced GSH content in pancreas of mice in AP group declined at each time point, and it decreased significantly at 3 h and 5 h (*p* < 0.05), and those in HAP group increased significantly at each time point when compared with the AP group (*p* < 0.05). The reduced GSH content in mice of HNS was lower than those of NS group significantly at 3 h and 5 h.

Compared with the NS group, the MDA content in pancreas of mice in AP group was significantly increased at 1 h, 3 h and 5 h (*p* < 0.05), and at each time point, the MDA content in pancreas of mice in HAP was significantly lower than those of the AP group (*p* < 0.05), as shown in Fig. 4b. While the pancreas MDA in HNS group was lower than those of NS group significantly at each time point (*p* < 0.05).

### Expression of Hsp60 protein in pancreatic tissue

**Fig. 5:**
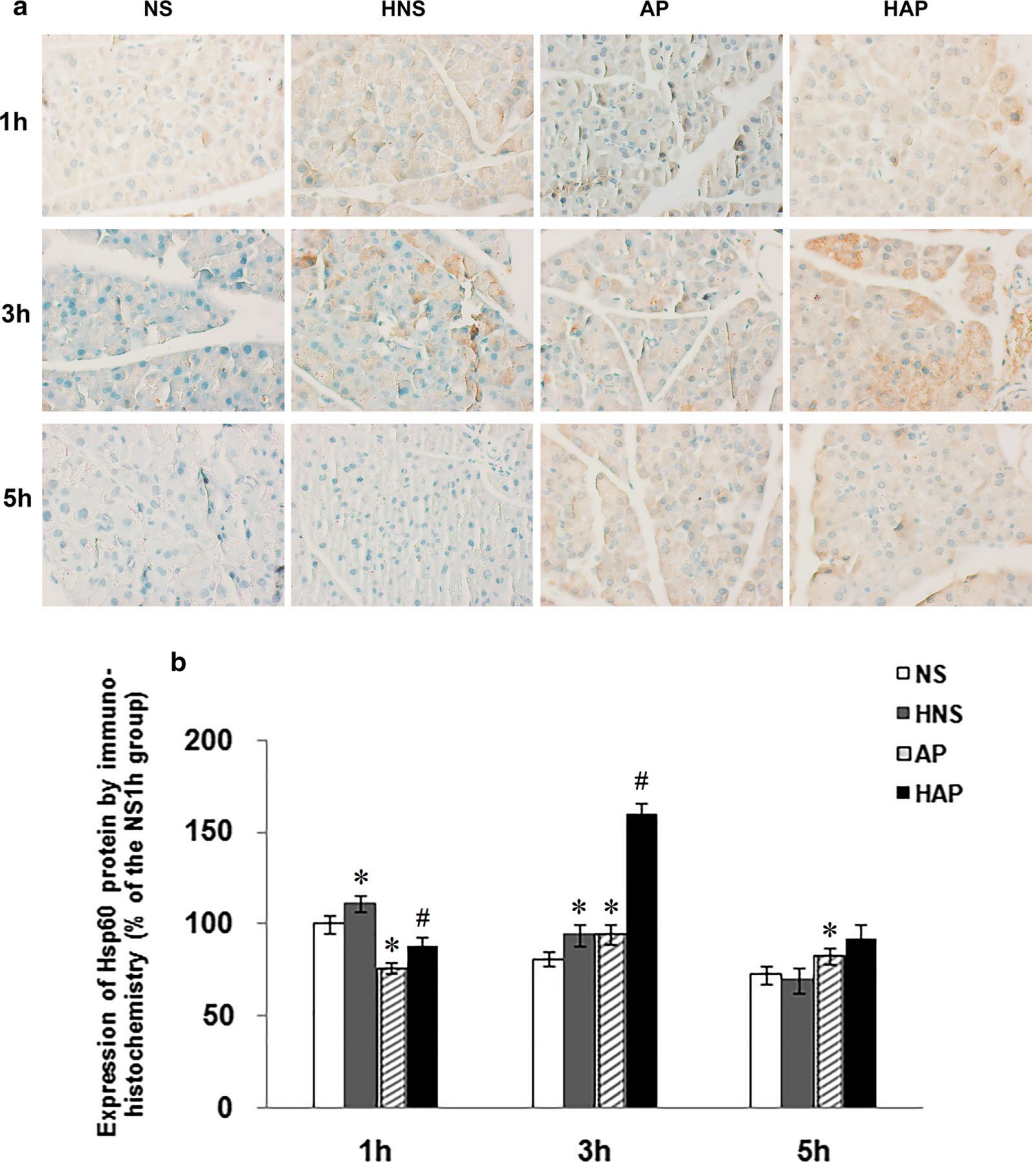
Effects of H_2_ pre-inhalation on expression of pancreatic Hsp60 protein by immunohistochemisty (× 400). **a** Representative images of Hsp60 protein expression detected by immunohistochemistry in pancreatic tissues; **b** Results of semi-quantitative analysis of the positive signals. Results are presented as means ± SEM (n = 3). *p < 0.05 compared with the NS group at the same time point; ^#^*p* < 0.05 compared with the AP group at the same time point

In situ Hsp60 protein expression in pancreas by immunohistochemistry was shown in Fig. 5. The brownish staining in Fig. 5a represented the expression of Hsp60 protein in the parenchyma of mouse pancreatic tissue. Average OD of the positive staining was show in Fig. 5b. It was found that the positive signals of Hsp60 protein in AP group was less than that in NS group at 1 h, and higher than that in NS group at 3 h and 5 h. Compared with the AP group, the positive signals in HAP group increased significantly at 1 h and 3 h. And compared with the NS group, it was increased at 1 h and 3 h in the HNS group.

**Fig. 6:**
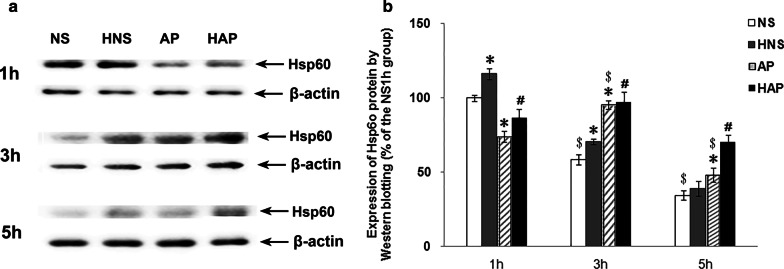
Effects of H_2_ pre-inhalation on expression of pancreatic Hsp60 protein by Western blotting. a, Representative images of Hsp60 protein expression detected by Western blotting in pancreatic tissues; b, Average integrated density of the positive signals. Results are presented as means ± SEM (n = 3). *p < 0.05 compared with the NS group at the same time point; ^#^*p* < 0.05 compared with the AP group at the same time point; ^$^*p* < 0.05 compared with the same group at 1 h time point

The expression of Hsp60 protein in mouse pancreas was checked by Western blotting, as shown in Fig. 6. It was also found that the expression of Hsp60 protein in pancreas of mice in AP group was less than that in NS group at 1 h, and higher than that in NS group at 3 h and 5 h. After the H_2_ treatment, the Hsp60 protein expression in HAP group increased significantly when compared with that in AP group at 1 h and 5 h. And the Hsp60 expression in HNS group was significantly higher than that in the NS group at 1 h and 3 h.

### Expression of Hsp60 mRNA in pancreatic tissue

**Fig. 7:**
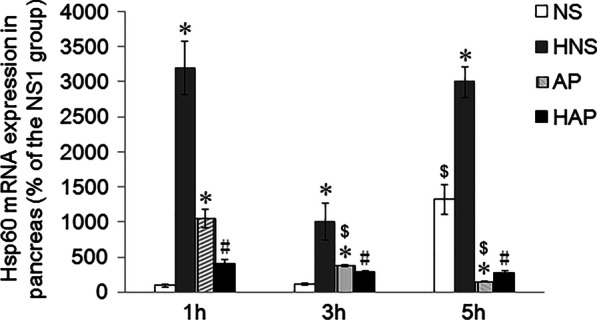
Effects of H_2_ pre-inhalation on expression of Hsp60 mRNA in pancreatic tissue. The expression of Hsp60 mRNA in pancreatic tissues was detected by real-time quantitative PCR and GAPDH as the house-keeping gene. Relative quantification was calculated by the double ΔCt method, and data were present as a percentage of the NS1h group. Results are presented as means ± SEM (n = 4). *p < 0.05 compared with the NS group at the same time point; ^#^*p* < 0.05 compared with the AP group at the same time point; ^$^*p* < 0.05 compared with the same group at 1 h time point

As shown in Fig. 7, the expression of Hsp60 in pancreas of mice in NS group increased over time and that in AP group decreased, especially at 5 h. Compared to the NS group, the expression of Hsp60 mRNA in AP group increased at 1 h and 3 h but decreased at 5 h significantly. Compared to the NS group, there was a big rise to the expression of Hsp60 mRNA in HNS group at each time point (p < 0.05). But the treatment failed to increase the Hsp60 mRNA at 1 h and 3 h, and it is only at 5 h that the expression of Hsp60 mRNA in HAP group higher than that in AP group significantly (p < 0.05).

## Discussion

Acute pancreatitis has been studied for over 100 years. The pathogenesis of the disease is not fully understood, and there are still many difficulties in the prevention and treatment of the disease. It is generally recognized that the premature activation of pancreatic enzymes in pancreatic acinar cells is an early and critical event in AP [11, 12]. Elevated plasma amylase and /or lipase, like the typical pathological change of pancreatic tissue and the indicators of inflammatory response, are important basis for clinical diagnosis of AP [2]. But the priming mechanism of pancreatic enzyme intracellular activation is far from being elucidated [1].

Pancreas is the second largest digestive gland in the human body and has a powerful exocrine function, and it can produce, store and secrete a large number of enzymes with powerful digestive functions. Normally, the various digestive enzymes of the pancreas are stored in zymogen granules of pancreatic acinar cells in an inactive way, secreted through the pancreatic duct into the intestinal cavity, activated by intestinal kinases and the alkaline environment, and play roles in food digestion. Studies have shown that the ordered synthesis, transport, modification and secretion of pancreatic enzymes are closely related to Hsp60 [3, 13]. And there are reports to reveal the protective effects of Hsp60 on AP, that is, water immersion stress induced Hsp60 expression protects against pancreatitis in rats [14], and down-regulation of Hsp60 worsen the injuries of isolated rat pancreatic tissues [5]. However, few studies have observed the protective effect of Hsp60 on AP in mice, and the mechanism of Hsp60 in the pathogenesis and protection of AP need further investigation[15].

Cerulein is a cholecystokinin analogue, and the mouse AP model induced by caerulein is classical and widely used animal AP model [16]. In the present study, caerulein induced AP mouse model was induced successfully, with typical pathological change of the pancreatic tissues, elevated amylase and lipase activity, and increased plasma IL-1 and IL-6 levels in AP mice as described above. Based on this AP model, it is observed that the expression of Hsp60 protein in control group decreased over time, but it in AP group show a different trend. At 1 h, the expression of Hsp60 protein in pancreatic tissue of AP group is significant lower than that of NS group, and at 3 h, the expression of Hsp60 protein in AP group increased significantly when compared with that at 1 h, and also higher than that of the NS group at 3 h, but at 5 h, it was significantly lower than that of the NS group. This may be explained by the fact that the injection at the time of modeling itself is a stress, and the increased expression of the induced Hsp60 is involved in the early protection of mice. The expression of Hsp60 in the AP group did not increase in the early stage, is this correlated to the occurrence of AP? Perhaps, the increase of the pancreatic Hsp60 protein in the AP group could not support the changes induced by the stress of caerulein damage, for example, it is not enough to accommodate the increase in the abnormal pancreatic enzymes.

There is another interesting finding in this study. The H_2_ treatment significantly increases the Hsp60 protein in the pancreas of the AP group, and more importantly, it simultaneously provides good protection against the caerulein-induced mouse AP, manifested by pathological changes, attenuated amylase and lipase activities, and decreased IL-1 and IL-6 levels. This is the first time to find that hydrogen treatment significantly improve the Hsp60 protein in mouse pancreatic tissues. Paradoxically, expression of Hsp60 mRNA in the pancreatic tissues in this study was not consistent with that of Hsp60 protein, and Strowski also reported inconsistent mRNA and protein expression of heat shock protein 70 in caerulein induced rat AP mode [17]. Here, we strongly agree with their view that failure to appropriately increase Hsp protein levels in response to high doses of caerulein might be a factor in the pathogenesis of pancreatitis [17]. The unsynchronous expression of Hsp60 mRNA and Hsp60 protein is interesting issue and need further investigation and discussion in the future, which might aid our understanding of the molecular mechanisms underlying caerulein-mediated pancreatitis. Now, it can be boldly speculated that an early increased Hsp60 protein expression might be partly contribute to the protective effect of hydrogen-rich gases on the acute AP.

Role of hydrogen in medicine has been concerned since 1975 [18], and it is generally accepted as a potential oxygen free radicals scavenger [6–9]. Recently, several works have found that hydrogen has a protective effect on AP, mainly due to its antioxidant effects [7–9]. Ren’s work is based on caerulein induced AP model in Balb /c mice and treated with hydrogen-rich saline after the modeling [7], and Zhou and Han’s work is based on AR42J cells treated with hydrogen-rich medium, and /or based on AP rat model treated with H_2_-rich gas after the modeling [8, 9]. The present work is based on caerulein induced AP model in C57Bl/6 mice, pre-treated with 3 days of hydrogen-rich gases before the first injection of the modeling, and further demonstrated the good protective effect of hydrogen on AP. Oxidative stress is a factor involved in the pathogenesis of AP, although the specific mechanism remains controversial [19, 20]. Therefore, pancreatic GSH and MDA which indicate the redox status and the oxidative damage in pancreatic tissues were also observed. Results shown H_2_ treatment increase pancreatic GSH and decrease pancreatic MDA in AP mice, indicating that antioxidant effects may be also involved in the protective mechanisms of the 3 days of hydrogen-rich gases inhalation on caerulein-induced AP mice.

Now we can have the idea that hydrogen has a protective effect on AP, and the antioxidant effects and Hsp60 expression may be both involved in the protective mechanism. Here, is there any possible correlation between the Hsp60 expression and the oxidative stress? Hsp60 is mitochondrial-derived chaperonin. Studies have shown that siHsp60 lead to the increase of oxidized protein in renal tubular cells [21]. Ren’s work ever show that hydrogen-rich saline inhibit pyrindomain-containing 3 (NLRP3) inflammasome activation and attenuates experimental mice AP [7], whereas report also show that Hsp60 can stimulate NLRP3 inflammasome pathway [22]. This suggests that increased the expression of Hsp60 protein may improve the oxidative stress. In the present study, both the increase of Hsp60 protein and the antioxidant activity were found in the protective effect of H_2_ on AP, their relationships and detailed mechanism need further investigation, including the effect of hydrogen-rich gases on other animal models of acute pancreatitis, and the comparative studies with conventional antioxidants.

## Conclusions

In conclusion, pre-inhalation of hydrogen-rich gases have a good protective effect on AP mice. Expression of Hsp60 in the AP group did not increase in the early stage. The pre-inhalation of hydrogen-rich gases increase the Hsp60 protein in pancreatic tissues, which may play role in the protective effects. Meanwhile, the antioxidant role of hydrogen may also be involved in the protective effects of hydrogen on AP mice. The relationship between Hsp60 and oxidative stress in the protective role of pre-inhalation of hydrogen-rich gases, and issue of the unsynchronous expression of Hsp60 mRNA and Hsp60 protein need further investigation. These works contributes to further elucidating the protective effects of hydrogen on acute pancreatitis, and also be of help in the understanding acute pancreatitis mechanism.

## Data Availability

Datasets are available through the corresponding author upon reasonable request.

## Abbreviations

AP: Acute pancreatitis
Hsp60: Heat shock protein 60
i.p.: Intraperitoneal injections
NS: Normal saline
HE: Hematoxylin and eosin
OD: Optical density
IL: Interleukin
ELISA: Enzyme-linked immune-sorbent assay
GSH: Reduced glutathione
MDA: Malondialdehyde
T-GSH: Total glutathione
GSSG: Oxidative glutathione
DNTB: Dithio-bis-nitrobenzoic acid
TBA: Thiobarbituric acid
BSA: Bovine serum albumin
DAB: Diaminobenzidine
HRP: Horse radish peroxidase
IntDen: Integrated density
RT-PCR: Real-time quantitative PCR
cDNA: Complementary DNA
SEM: Standard error of the mean
